# Comparison of efficacy and adverse effects of CD19/20 CART versus CD19 single-target CART in R/R DLBCL: a single-center retrospective study

**DOI:** 10.3389/fimmu.2025.1582944

**Published:** 2025-05-06

**Authors:** Bin Xue, Yifan Liu, Bing Li, Yan Lu, Lili Zhou, Shiguang Ye, Huina Lu, Xiu Luo, Aibin Liang, Ping Li

**Affiliations:** ^1^ Department of Hematology, Shanghai Tongji Hospital, Tongji University School of Medicine, Shanghai, China; ^2^ Clinical Research Ward of Cancer Center, Shanghai Tongji Hospital, Tongji University School of Medicine, Shanghai, China

**Keywords:** CAR-T, lymphoma, dual-target, CD19, CD19/20

## Abstract

**Purpose:**

CD19 Chimeric Antigen Receptor T-cell therapy (CART) represents a groundbreaking approach in the treatment of relapsed or refractory diffuse large B-cell lymphoma (R/R DLBCL). However, a subset of patients fails to achieve optimal outcomes with CD19-targeted CAR T-cells alone. To address these limitations, the development of multi-targeted CART therapies has become a focal point of innovative research. This study aims to compare the therapeutic efficacy and adverse events of dual-target versus single-target CART therapies in R/R DLBCL patients through a single-center retrospective analysis.

**Methods:**

We included 70 patients with R/R DLBCL treated at Shanghai Tongji Hospital between January 1, 2019, and December 31, 2021. Among them, 20 patients received dual-target (CD19/20) CART, while 50 underwent CD19 CART.

**Results:**

The CD19/20 CART group demonstrated significantly superior three-month efficacy to the CD19 CAR T-cell group, with a notably higher complete response (CR) rate. The median progression-free survival (PFS) and overall survival (OS) were 28.6 and 31.8 months longer in the Bi-CART group compared to the CD19 CAR T-cell group. However, the two groups had no significant differences in overall PFS, duration of response (DOR), or OS. The CD19/20 CART group exhibited a higher incidence of cytokine release syndrome (CRS), hematological toxicity, infections, and secondary primary tumors.

**Conclusion:**

This study highlights the superior efficacy of dual-target CAR T-cell therapy in managing R/R DLBCL patients. The dual-target therapy significantly extended median survival compared to CD19 single-target CAR T-cell therapy. However, the enhanced therapeutic benefits were accompanied by a higher incidence of adverse effects.

## 1 Introduction

Diffuse large B-cell lymphoma (DLBCL) is the most common form of non-Hodgkin lymphoma (NHL), accounting for approximately 50% of newly diagnosed B-cell lymphomas globally. (1) The primary therapeutic modalities for DLBCL include chemotherapy, radiation, and stem cell transplantation, with R-CHOP (rituximab, cyclophosphamide, doxorubicin, vincristine, and prednisone) being the established standard regimen, though 30-40% of patients still experience relapse or develop resistance to therapy. (2–4) Despite significant advancements, some patients experience treatment failure, leading to relapsed or refractory (R/R) DLBCL, which has a poor prognosis due to the absence of standardized, globally effective treatments.

Chimeric Antigen Receptor T-cell therapy (CART) marks a revolutionary breakthrough in the treatment of DLBCL, offering new hope for patients who have failed conventional therapies. The landmark ZUMA-1 trial reported a complete response rate of approximately 58%, a notable achievement given the poor prognosis with advanced DLBCL. (5, 6) Consistent with these findings, the JULIET and TRANSCEND trials have also demonstrated significant response rates, further underscoring the potential of this therapy. (7, 8) Despite these encouraging results, several challenges remain. Over 60% of patients experience relapse, often due to antigen escape—a phenomenon in which tumor cells lose or alter the expression of the target antigen (CD19)—thereby diminishing the efficacy of CAR T-cell therapy and contributing to disease recurrence (5, 9, 10). Additionally, resistance to subsequent CAR T-cell treatments can develop, underscoring the critical need for further innovations, such as multi-antigen targeting or strategies to enhance T-cell persistence, which are essential (11).

The development of dual-targeting strategies, particularly those addressing both CD19 and CD20 antigens, represents a promising advancement in overcoming antigen escape in CAR T-cell therapy. (12) This approach involves genetically engineering T-cells to either express two separate CAR molecules or a single bispecific CAR capable of recognizing both CD19 and CD20. (13) Early studies have indicated that this approach not only mitigates antigen heterogeneity but also enhances the durability of therapeutic responses, marking a critical step forward in addressing the limitations of single-target therapies. (14) A recent phase 1/2 trial conducted at our center demonstrated encouraging response rates and an acceptable safety profile, underscoring the potential of dual-target CAR (CD19/20) T-cell therapy in patients with refractory or relapsed DLBCL. These findings highlight the promise of this innovative approach in addressing treatment resistance and improving outcomes for this challenging patient population (15, 16).

To date, no rigorous Randomized Controlled Trials (RCT) have been conducted to directly compare the efficacy and safety of the two CART treatments. To address this gap, we conducted a single-center retrospective study to compare the therapeutic efficacy of dual-target versus single-target CART therapies in R/R DLBCL patients and to assess differences in the incidence and types of adverse events associated with each treatment.

## 2 Methods

### 2.1 Study population and design

Data from 70 patients diagnosed with R/R DLBCL, who received either CD19 CART or CD19/20 CART (Bi-CART), were retrospectively collected from Shanghai Tongji Hospital between January 1, 2019, and December 31, 2021. All patients exhibited stable disease (SD) or progressive disease (PD) status prior to undergoing cell therapy. This cohort included one case of primary mediastinal large B-cell lymphoma (PMBCL) and one case of follicular lymphoma that had histologically transformed into diffuse large B-cell lymphoma (tFL-DLBCL). The study protocols were approved by the Ethics Committee of Shanghai Tongji Hospital, Tongji University, and were conducted in compliance with the principles of the Declaration of Helsinki. All patients provided written informed consent.

### 2.2 Inclusion and exclusion

The CD19 CART group came from 4 different clinical trials, all of which used 4-1BB for costimulation (NCT02537977, NCT03154775, CTR20201986, CTR20200561). CD19/20 CART (Prizloncabtagene autoleucel, Prizlon-cel, C-CAR039) has been developed as a novel 2nd generation 4-1BB bi-specific CAR-T targeting both CD19 and CD20 antigens with an optimized bi-specific antigen binding domain, from clinical trial NCT04317885. In these clinical trials, patients aged 18–75 years who voluntarily participated and signed informed consent were included in the study. Inclusion of CD19 or CD20 positive DLBCL (including PMBCL and tFL) confirmed by cytology or histology according to 2016 WHO criteria. (17) For CD20-positive subjects, they should have received at least one regimen containing anti-CD20-targeted therapy (such as rituximab). And one follicular lymphoma patient and one primary central nervous system lymphoma patient were excluded.

### 2.3 Definitions of therapy and efficacy

The drug-eluting period between bridging therapy and initiating lymphodepletion for CAR-T therapy strictly adhered to the practice recommendations jointly issued by the European Society for Blood and Marrow Transplantation (EBMT) and the Joint Accreditation Committee of ISCT and EBMT (JACIE) and the European Haematology Association (EHA) (18).

In accordance with the guidelines established by the U.S. Food and Drug Administration, the lymphodepletion regimen consisted of fludarabine at a dose of 25 mg/(m²*d) from day -5 to -3, and cyclophosphamide at 300 mg/(m²*d) from day -5 to -3, prior to the infusion of CD19 CAR/Bi-CAR T-cells. (7, 19, 20) The total dose of CAR-T cells ranged from 1-5^10^6^/kg. The occurrence and severity of cytokine release syndrome (CRS) and immune effector cell-associated neurotoxicity (ICANS) were documented and graded on the basis of the consensus guidelines provided by the American Society of Transplantation and Cellular Therapy (ASTCT) (21).

The Lugano classification (2014) was utilized to assess the response following the infusion of CD19 CAR T-cells and Bi-CAR T-cells. This classification relies on CT and PET-CT scans to evaluate the treatment response. (22) The response categories included complete response (CR), partial response (PR), stable disease, and progressive disease. The overall objective response (ORR) was evaluated based on the best response (CR+PR) within 3 months following CAR T-cells infusion. Progression-free survival (PFS) was defined as the interval from CAR T-cell infusion until disease progression, death from any cause, or the date of the last follow-up visit, whichever occurred first. Duration of response (DOR) referred to the time from the first assessment of CR or PR after CAR T-cell infusion to the first occurrence of disease progression or death from any cause, whichever came first. Overall survival (OS) was measured from the time of CAR T-cell infusion until death from any cause or the date of the last follow-up visit, whichever occurred first. And the follow-up period concluded on September 30, 2024.

According to the 2016 World Health Organization (WHO) classification for tumors of hematopoietic and lymphoid tissues, patients were diagnosed with DLBCL based on pathological evaluation. The classification of double-expressor lymphoma (characterized by overexpression of MYC and BCL-2 proteins) and double/triple-hit lymphoma (involving MYC and BCL2 and/or BCL6 rearrangements) followed standard diagnostic criteria. (17) The cell of origin (COO) classification, distinguishing between germinal center B-cell (GCB) and non-GCB subtypes, was determined using the Hans algorithm. (23) TP53 alterations were identified through mutations detected by next-generation sequencing (NGS) (24) or deletions observed via fluorescence *in situ* hybridization (FISH) analysis, based on the most recent pathological test conducted prior to CAR-T therapy (25).

### 2.4 Statistical analysis

The statistical analysis in this study was conducted primarily via R software (version 4.3.2, Boston, Massachusetts, USA)R Core Team (2022). R: A language and environment for statistical computing. R Foundation for Statistical Computing, Vienna, Austria. URL https://www.R-project.org/.12c, SPSS software (version 22.0, Chicago, Illinois, USA), and GraphPad Prism software (version 8.0.1). In this study, the normality test of continuous variables was performed via the Kolmogorov–Smirnov test. The Levene test was conducted for continuous variables with a normal distribution to assess homogeneity of variance. If there was no violation of the assumption of homogeneity of variance (P≥0.05), the data were reported as the mean standard deviation and analyzed via one-way ANOVA. Continuous variables with violated homogeneity of variances or nonnormal distributions are reported as medians edinterquartile ranges (IQR) and were analyzed via the Kruskal-Wallis test. The expected frequency of the cross-category in the categorical variable is not less than 5, the chi-square test is used for analysis. If the expected frequency was less than 1, then Fisher’s exact test was used for analysis. Given the exploratory nature of this retrospective study and the limited availability of CAR-T recipients during the study period, formal sample size calculation was not performed *a priori*. *Post-hoc* power analysis using the G*Power 3.1 (Z-test, two-tailed) demonstrated exceptional achieved power (98.8%) at α=0.05. (26) PFS, DOR and OS were visualized via Kaplan–Meier curves. The reported p values were two-sided, P<0.05 considered a statistically significant result.

## 3 Results

### 3.1 Baseline information

The median age of patients receiving CD19 CART therapy was 59.0 years (IQR: 49.3-67.0), the median follow-up date was 48.9 months (IQR: 40.13-58.6). While that of patients in the Bi-CART group was 58.5 years (IQR: 50.3-63.5), the median follow-up date was 52.5 months (IQR: 48.2-54.2), the median age between the two groups and the median follow-up date had no statistical difference. Baseline characteristics were well balanced between the two groups (**Table 1**), including general demographics [gender, age, Eastern Cooperative Oncology Group (ECOG) performance status prior to infusion], disease stage (Ann Arbor stage, proportion of extranodal disease), tumor burden [International Prognostic Index (IPI) score, percentage of bulky disease, lactate dehydrogenase (LDH) levels before infusion], tumor characteristics (Hans classification, double expression, double/triple-hit status, TP53 abnormalities), and prior treatments (percentage of prior ASCT, prior radiotherapy, and number of previous therapy lines). No significant differences were observed between the groups (P>0.05). It is worth noting that data on TP53 abnormalities were incomplete, as five patients in the Bi-CART group did not undergo NGS or FISH testing on their pathological biopsies.

**Table 1:** Baseline table.

Characteristics	Overall, N = 70	CD19 CART, N = 50	CD19/20 CART, N = 20	P-value
Gender, n (%)				0.496
Male	36 (51)	27 (54)	9 (45)	
Female	34 (49)	23 (46)	11 (55)	
Age at enrollment, n (%)				>0.999
<60y	35 (50)	25 (50)	10 (50)	
≥60y	35 (50)	25 (50)	10 (50)	
Hans classification, n (%)				0.676
GCB	20 (29)	15 (30)	5 (25)	
N-GCB	50 (71)	35 (70)	15 (75)	
Double expression, n (%)				0.280
Yes	28 (40)	22 (44)	6 (30)	
No	42 (60)	28 (56)	14 (70)	
Double/triple-hit, n (%)				0.067
Yes	4 (6)	1 (2.0)	3 (15)	
No	66 (94)	49 (98)	17 (85)	
Ann Arbor stage, n (%)				0.708
I-II	9 (13)	6 (12)	3 (15)	
III-IV	61 (87)	44 (88)	17 (85)	
ECOG before infusion, n (%)				0.597
0	35 (50)	24 (48)	11 (55)	
1	35 (50)	26 (52)	9 (45)	
IPI score at enrollment, n (%)				0.597
0-2	35 (50)	24 (48)	11 (55)	
3-4	35 (50)	26 (52)	9 (45)	
Bulky disease, n (%)				0.680
<7.5 cm	62 (89)	45 (90)	17 (85)	
≥7.5 cm	8 (11)	5 (10)	3 (15)	
Extra-nodual disease, n (%)				0.395
0-1	19 (27)	15 (30)	4 (20)	
≥2 organs	51 (73)	35 (70)	16 (80)	
TP53, n (%)				0.373
*(Missing)*	5 (7)	0 (0)	5 (25)	
WT	41 (59)	33 (66)	8 (40)	
Altered	24 (34)	17 (34)	7 (35)	
Prior ASCT, n (%)				0.708
Yes	9 (13)	6 (12)	3 (15)	
No	61 (87)	44 (88)	17 (85)	
Prior radiotherapy, n (%)				0.931
Yes	18 (26)	13 (26)	5 (25)	
No	52 (74)	37 (74)	15 (75)	
Prior lines of therapy, n (%)				0.194
1-3	30 (43)	19 (38)	11 (55)	
≥4 lines	40 (57)	31 (62)	9 (45)	
LDH level before infusion, n (%)				0.290
≤ULN (%)	35 (50)	27 (54)	8 (40)	
>ULN (%)	35 (50)	23 (46)	12 (60)	

In the CD19 CART group, 13 patients (26.0%) received targeted drugs with chemotherapy as bridging therapy, and 2 patients (4.0%) received maintenance therapy with targeted drugs as part of their bridging protocol. In the Bi-CART group, 4 patients (20.0%) received targeted drugs with chemotherapy as bridging therapy.

### 3.2 Efficacy of CAR-T therapy

**Figure 1:**
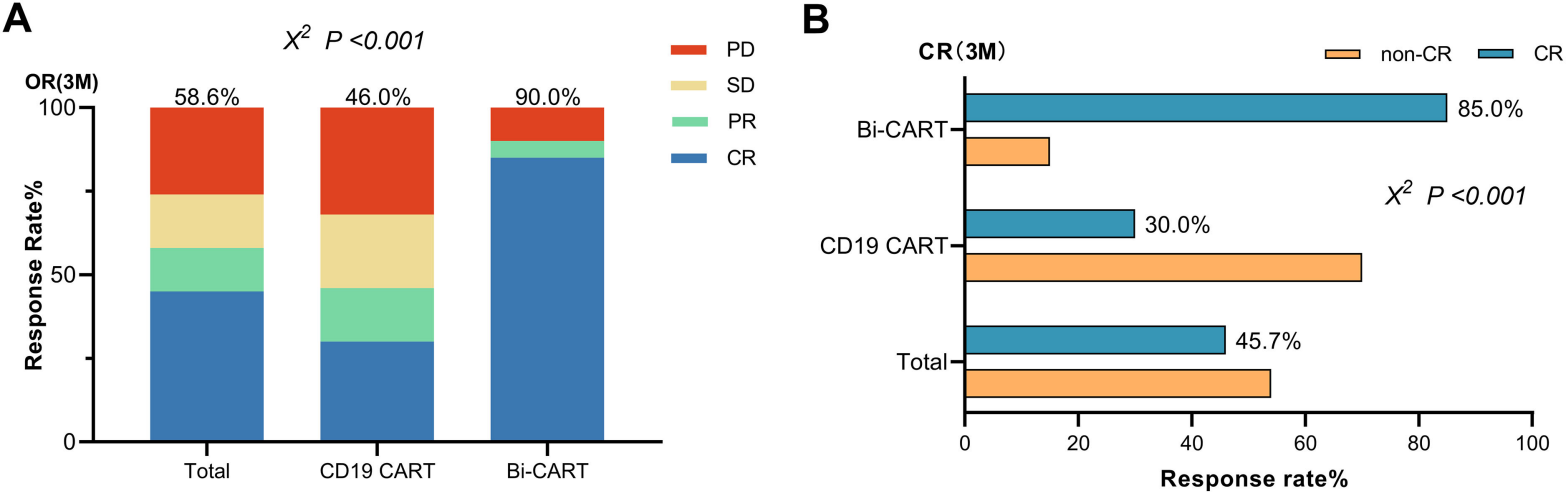
Efficacy of contrast in the CD19 CART and Bi-CART groups. **(A)** Best response (CR+PR) within 3 months of CARTs infusion (P<0.001). **(B)** The CR rate of the two groups within 3 months of CARTs infusion (P<0.001).

The cohort comprised 56 progressive disease and 14 stable disease patients at baseline (**Supplementary Figure 1**). While dual-target CAR-T recipients had numerically higher PD prevalence (95% vs. 74%, P=0.054), pretreatment status showed no association with ORR (P=0.903) or CR rates (CRR, P=0.719) in regression models. Subgroup analyses by treatment type confirmed consistent response patterns regardless of baseline disease status (**Supplementary Table 1**). Among patients who achieved ORR within 3 months of infusion (**Figure 1A**), 23 (46.0%) were from the CD19 CART group, and 18 (90.0%) were from the Bi-CART group, with the difference being statistically significant (P<0.001). CRR were 15 (30.0%) and 17 (85.0%) in the CD19 CART and Bi-CART groups (**Figure 1B**), respectively, also showing a statistically significant difference (P<0.001).

**Figure 2:**
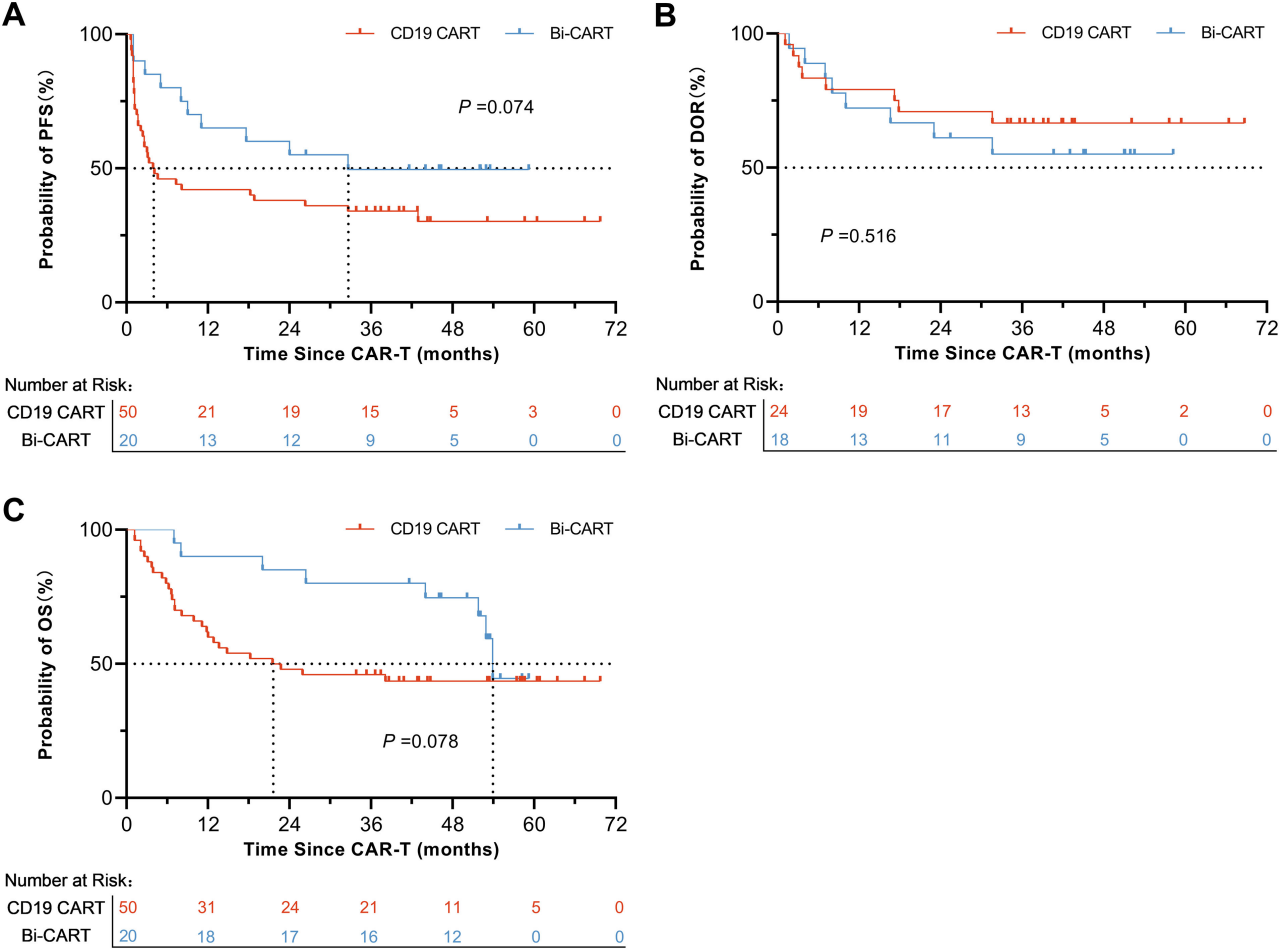
Prognosis of contrast in the CD19 CART and Bi-CART groups. Survival of patients with r/r DLBCL treated with CD19 CAR-T, **(A)** PFS (log-rank test P=0.074), **(B)** DOR (log-rank test P=0.516) and **(C)** OS (log-rank test P=0.076).

The proportion of patients suffering disease recurrence after treatment was 34 (68.0%) in the CD19 CART group and 10 (50.0%) in the Bi-CART group, with no statistically significant difference (P=0.159). Baseline characteristics of IPI score ≥3 (P<0.001) and TP53 abnormalities (P=0.007) as predictors of disease relapse (**Supplementary Table 2**), with no significant treatment-subgroup interactions detected (all P for interaction >0.1, **Supplementary Table 3**). The median PFS times were 4.0 months [95% Confidence Interval (CI): 2.4-42.9] and 32.6 months (95% CI: 11.0-Not Reach) for the CD19 CART and Bi-CART groups, respectively. The log-rank test for the PFS Kaplan-Meier curve yielded a statistic of 3.18, Hazard Ratio (HR) =0.54 (95% CI: 0.29-1.00), with a p-value of 0.074, indicating no statistically significant difference (**Figure 2A**). Within 3 months of achieving OR, there were 8 cases of recurrence (33.3%) in the CD19 CART group and 8 cases (44.4%) in the Bi-CART group, with no statistically significant difference (P=0.463). The median DOR in both groups was not reached (NR). The log-rank test for the DOR Kaplan-Meier curve produced a statistic of 0.42, HR = 1.38 (95% CI: 0.51-3.73), with a P-value of 0.516, indicating no statistically significant difference (**Figure 2B**). Deaths occurred in 28 patients (56.0%) in the CD19 CART group and 8 patients (40.0%) in the Bi-CART group, with no statistically significant difference (P=0.226). The median OS times were 22.1 months (95% CI: 11.8-NR) for CD19 CART and 53.9 months (95% CI: 51.8-NR) for Bi-CART. The log-rank test for the OS Kaplan-Meier curve yielded a statistic of 3.10, HR = 0.50 (95% CI: 0.25-0.99), with a P-value of 0.078, indicating no statistically significant difference (**Figure 2C**). While the extension of OR and median PFS within 3 months suggests an enhanced disease response, the depth of response remains uncertain due to recurrent events, relapses after achieving OR, and the lack of a difference in DOR.

All deaths in the CD19 CART group were due to disease progression. In the Bi-CART group, two patients died from causes unrelated to primary disease progression: one from acute myeloid leukemia (AML) at 26.4 months and another from a cerebrovascular accident at 40.8 months.

### 3.3 Adverse reactions of CAR-T therapy

CRS occurred in 32 patients (64.0%) in the CD19 CART group and 18 patients (90.0%) in the Bi-CART group (**Table 2**), with a statistically significant difference (P=0.030). Grade 3–4 CRS was observed in 2 patients (4.0%) in the CD19 CART group, while no cases were reported in the Bi-CART group (P>0.999). The median time to CRS onset in the CD19 CART group was 5.5 days (IQR: 0.8-8.0 days), and the median duration was 5.5 days (IQR: 3.8-10.2 days). For the Bi-CART group, the median time (4.0 days, IQR: 2.0-7.8 days) to CRS onset was not significantly different (P=0.943), with a median duration of 6.5 days (IQR: 3.3-8.0 days, P= 0.887). Grade 1 ICANS was observed in 2 patients (4.0%) in the CD19 CART group and 1 patient (5.0%) in the Bi-CART group, with no statistically significant difference (P>0.999). In the CD19 CAR-T group, ICANS occurred on Day 5 (lasting 1 day) and Day 23 (lasting 4 days). In the Bi-CART group, ICANS occurred on Day 18 and lasted 2 days. All cases of CRS and ICANS were managed according to the ASTCT and 2022 Chinese consensus guidelines (27) using Nonsteroidal Anti-inflammatory Drugs (NSAIDs), Corticosteroids, and Tocilizumab, with no deaths occurring during these reactions. All events resolved completely under protocolized management; the occurrence, severity and management of CRS were not associated with clinical outcomes (**Supplementary Table 4**).

**Table 2:** Safety of CAR T-cell therapy.

Variable		CD19 CART, N = 50	CD19/20 CART, N = 20	P-value
CRS (grade), n (%)				0.030
0		18 (36)	2 (10)	
≥1	1-2	30 (60)	18 (90)	>0.999
	3-4	2 (4)	0 (0)	
ICANS (grade), n (%)				>0.999
0		48 (96)	19 (95)	
1		2 (4)	1 (5)	
Hematological toxicity, n (%)				
Anemia, n (%)	Yes	14 (28)	12 (60)	0.012
	No	36 (72)	8 (40)	
Thrombocytopenia, n (%)	Yes	20 (40)	15 (75)	0.008
	No	30 (60)	5 (25)	
Neutropenia, n (%)	Yes	40 (80)	17 (85)	0.744
	No	10 (20)	3 (15)	
Infections, n (%)				0.031
Yes		26 (52)	16 (80)	
No		24 (48)	4 (20)	
Primary secondary tumor, n (%)				0.079
Yes		0 (0)	2 (10)	
No		50 (100)	18 (90)	

After the infusion of CAR T-cells, anemia occurred in 14 patients (28.0%) in the CD19 CART group and 12 patients (60.0%) in the Bi-CART group, with a statistically significant difference (P=0.012). Thrombocytopenia occurred in 20 patients (40.0%) in the CD19 CART group and 15 patients (75.0%) in the Bi-CART group, with a significant difference (P=0.008); Neutropenia occurred in 40 patients (80.0%) in the CD19 CART group and 17 patients (85.0%) in the Bi-CART group, with no significant difference (P=0.744). Infections occurred in 26 patients (52.0%) in the CD19 CART group and 16 patients (80.0%) in the Bi-CART group, showing a statistically significant difference (P=0.031). Two patients (10.0%) in the Bi-CART group developed secondary primary malignancies. One patient developed AML in the 10th month after treatment and later died. The other developed Epstein-Barr virus-positive cytotoxic T-cell lymphoma at 8 months, with no CAR transgene detected via tumor biopsy (qPCR). No cases of secondary malignancies were observed in the CD19 CART group (P=0.079).

### 3.4 Treatment following CAR-T therapy

**Figure 3:**
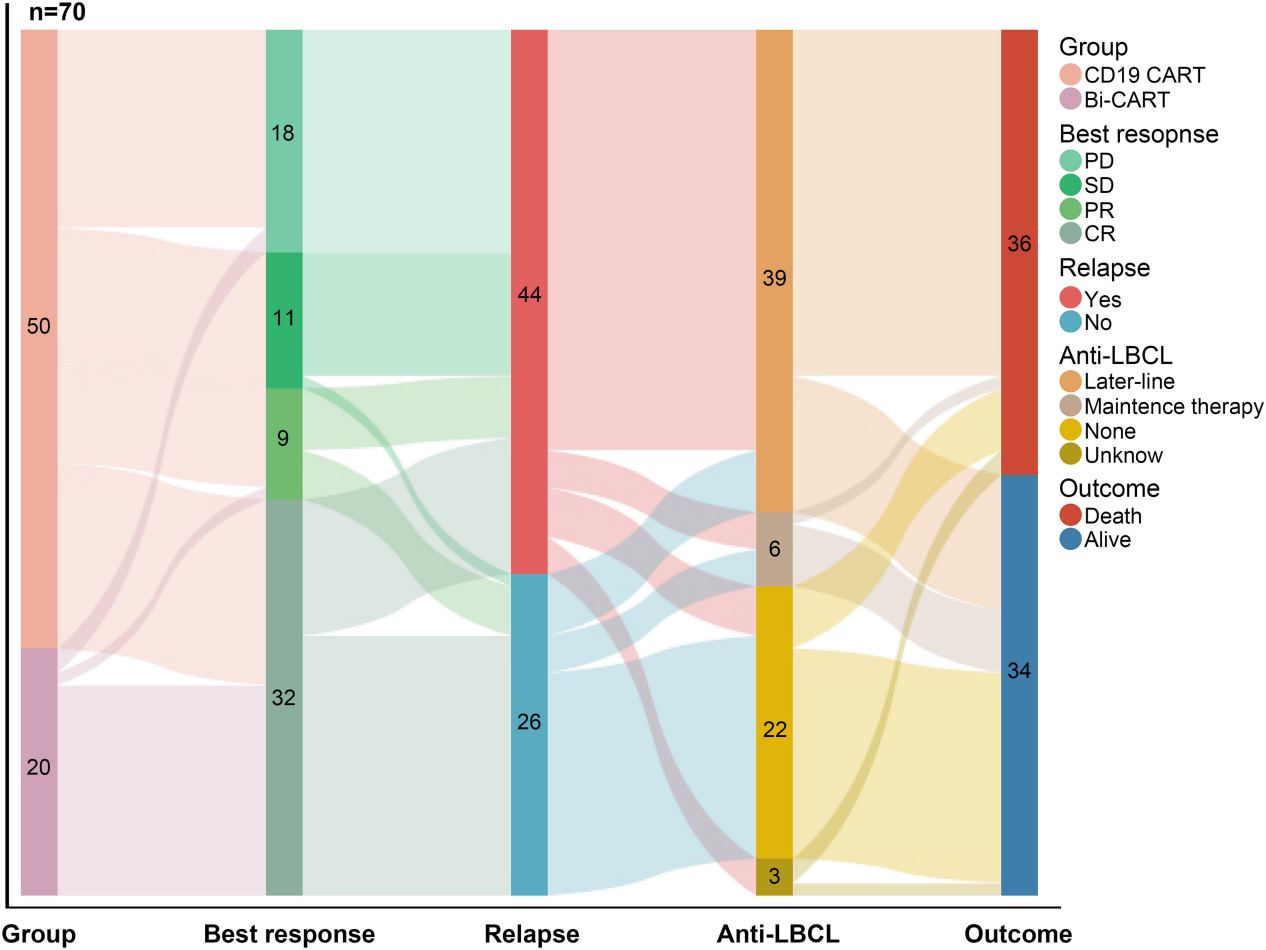
The treatment thread diagram for the entire study cohort. As of 2024/09/30, of the 70 patients, 41 achieved OR, 44 experienced disease recurrence, 45 received continued treatment, and 36 eventually died.

The treatment strategy for the entire study cohort is illustrated in the treatment thread diagram (**Figure 3**). All patients who experienced relapses within 3 months of CAR-T therapy, as well as those with subsequent relapses, received later-line therapy, except for 4 patients with ultra-rapid disease progression who only underwent life-sustaining treatment. As for later-line treatments, 25 patients received a new targeted immunotherapy combined with chemotherapy; 13 patients were treated with a novel CAR-T therapy, and 1 patient received a novel CAR-T therapy combined with autologous stem cell transplantation (ASCT); 6 patients received maintenance therapies, including immune checkpoint inhibitors (ICIs), Bruton’s tyrosine kinase (BTK) inhibitors, Lenalidomide, and Sidanidine. Follow-up information was missing for 3 patients who experienced disease progression after CAR-T therapy.

## 4 Discussion

CD19 CAR-T therapy has demonstrated remarkable advancements over traditional chemotherapy, offering new hope to patients with R/R DLBCL patients. (7, 19, 28–31) Despite these successes, our previous research indicates that CD19 CAR-T therapy remains ineffective for certain patients. (32) To overcome resistance and antigen escape in R/R DLBCL, research is focusing on durable strategies, including alternative antigen targeting (CD20, CD22, CD70), combination therapies, and next-generation CAR-T or bispecific antibodies (CD19/20, CD19/22, CD19/70) to expand treatment options for CD19-negative relapses. (33–38) Dual-target CAR-T therapy may outperform single-target approaches by broadening tumor cell elimination, enhancing T-cell activation, extending CAR-T persistence for long-term surveillance, and improving immune penetration to strengthen anti-tumor efficacy (39, 40).

Notably, only one two-arm study has been reported so far, and dual-target studies showed no significant improvement in complete response or recurrence rates compared to single-target studies. (41) Other existing research and review articles primarily compare patients across different studies, which lack methodological rigor. (40) For the first time, we conducted a retrospective comparison of single-target CD19 CART and dual-target CART in both arms with long-term follow-up of more than 5 years. Results indicate that CD19/20 CART therapy achieves superior short-term efficacy compared to CD19 CART, as evidenced by metrics such as the best response at 3 months, median PFS, and median OS. However, dual-target CART does not show a significant advantage in terms of long-term survival. The dissociation between early CR superiority and comparable long-term outcomes in dual-target therapy suggests distinct biological mechanisms governing initial response versus sustained remission. While enhanced antigen coverage may improve tumor clearance efficiency, TP53-driven genomic instability and high tumor burden appear to ultimately determine relapse risk through CAR-T-resistant clonal evolution (39, 40, 42, 43). A comparison between the DOR curves of the two groups could also confirm this argument, with CAR-T responders achieving sustained remission regardless of target configuration.

Integrating dual-target CAR-T therapy into current treatment regimens demands robust evidence of its safety profile. Clinical trials and real-world studies are essential to assess potential side effects, particularly CRS and ICANS. Previous studies have indicated that CD19 CAR-T therapy is an independent factor contributing to the development of CRS, potentially resulting in more severe levels of CRS. (41) Our study showed that CD19/20 CART had a higher probability of CRS, but there was no significant difference in the severity or duration of CRS between the two groups, nor was there a significant difference in the incidence of ICANS. Further analysis of CRS grade and treatment intensity was not associated with CART response. The dissociation between CRS incidence and severity highlights that risk-adapted management (27) can effectively mitigate severe toxicity while preserving anti-tumor efficacy.

Another point of note is that patients who received CD19/20 CART had a higher incidence of primary secondary tumor (2 in 20, although no statistical difference). The big data analysis of the FDA Adverse Events Reporting System sheds light on the increased reporting of myeloid neoplasms, T-cell lymphomas, and certain types of solid tumor after commercial CART in 2024 (4.3%, 536 of 12,394). Considering the imbalance of analysis and the low incidence, secondary tumor cannot be considered directly related to CART. (44) Case reports describe CART-associated T-cell lymphomas potentially linked to viral vector integration mutagenesis, though comprehensive genomic analyses suggest different characteristics rather than direct CART causality. (45, 46) In our study, the two secondary malignancies observed in CD19/20 CART recipients were not considered to be related to CART, because no CAR transgene was detected via tumor biopsy. At present, there are few clinical retrospective statistics on secondary tumors after dual-target CART, but some studies suggest that the design of dual-target CART may increase the risk of insertional mutagenesis (47) and replicative stress (48). Secondary tumor occurrence of single-/dual-target CART will be reported in further follow-up.

Several limitations of this study should be acknowledged. First, the single-center cohort and relatively small sample size may restrict the generalizability of our findings to broader populations, and unmeasured confounding factors inherent to retrospective designs cannot be fully excluded. While *post-hoc* analyses suggested sufficient statistical power (98.8%) to detect the observed ORR difference, larger prospective cohorts are needed to validate subgroup findings and long-term outcomes. Second, the inability to control post-relapse therapeutic heterogeneity, such as secondary CAR-T reinfusion or conventional salvage chemotherapy, prevents definitive assessment of how subsequent interventions modulate long-term survival outcomes. Third, inter-group CAR-T platform disparities (multi-trial CD19 products vs. uniform CD19/20 bi-specific constructs) introduce confounding from divergent manufacturing protocols and pharmacokinetic behaviors.

To address these limitations, future prospective multicenter randomized controlled trials are warranted. Such studies should prioritize (a) standardized patient stratification based on tumor burden, prior treatment lines, and molecular biomarkers; (b) protocol-defined allocation of relapse interventions (e.g., randomized assignment to secondary CAR-T or chemotherapy) with rigorous adjustment for baseline prognostic variables; and (c) harmonized therapeutic protocols across institutions to minimize inter-center variability. This will help clinicians select more precise and less harmful CAR-T therapy options for patients with relapsed/refractory disease.

## 5 Conclusion

For the first time, our study demonstrates that dual-target CAR T-cell therapy (CD19/20) achieves a superior therapeutic compared to single-target CAR T-cell therapy (CD19) in the treatment of R/R DLBCL, significantly extending median survival. However, it is associated with a higher incidence of adverse effects, including CRS, hematological toxicity, infections, and secondary primary tumors. While the integration of dual-target CAR T-cell therapy into the DLBCL treatment landscape holds great promise, further optimization is essential. This will help enhance tumor remission rates while mitigating adverse effects, ultimately improving patient outcomes.

## Data Availability

The raw data supporting the conclusions of this article will be made available by the authors, without undue reservation.
