# Supplementary material for: Comparison of efficacy and adverse effects of CD19/20 CART versus CD19 single-target CART in R/R DLBCL: a single-center retrospective study
Source: Front Immunol. 2025 May 6;16:1582944. doi: 10.3389/fimmu.2025.1582944 (PMC12089104; doi:10.3389/fimmu.2025.1582944)

**Supplementary Figure 1. Disease status before and after CART treatment (within 3 months).**

Pretreatment disease status distribution among the entire cohort (N=70): PD for 56 patients, SD for 14 patients (20.0%). Group stratification revealed: 37 PD (74.0%) vs. 13 SD (26.0%) for CD19 CA-T group, 19 PD (95.0%) vs. 1 SD (5.0%) for CD19/20 CART group, P=0.054.


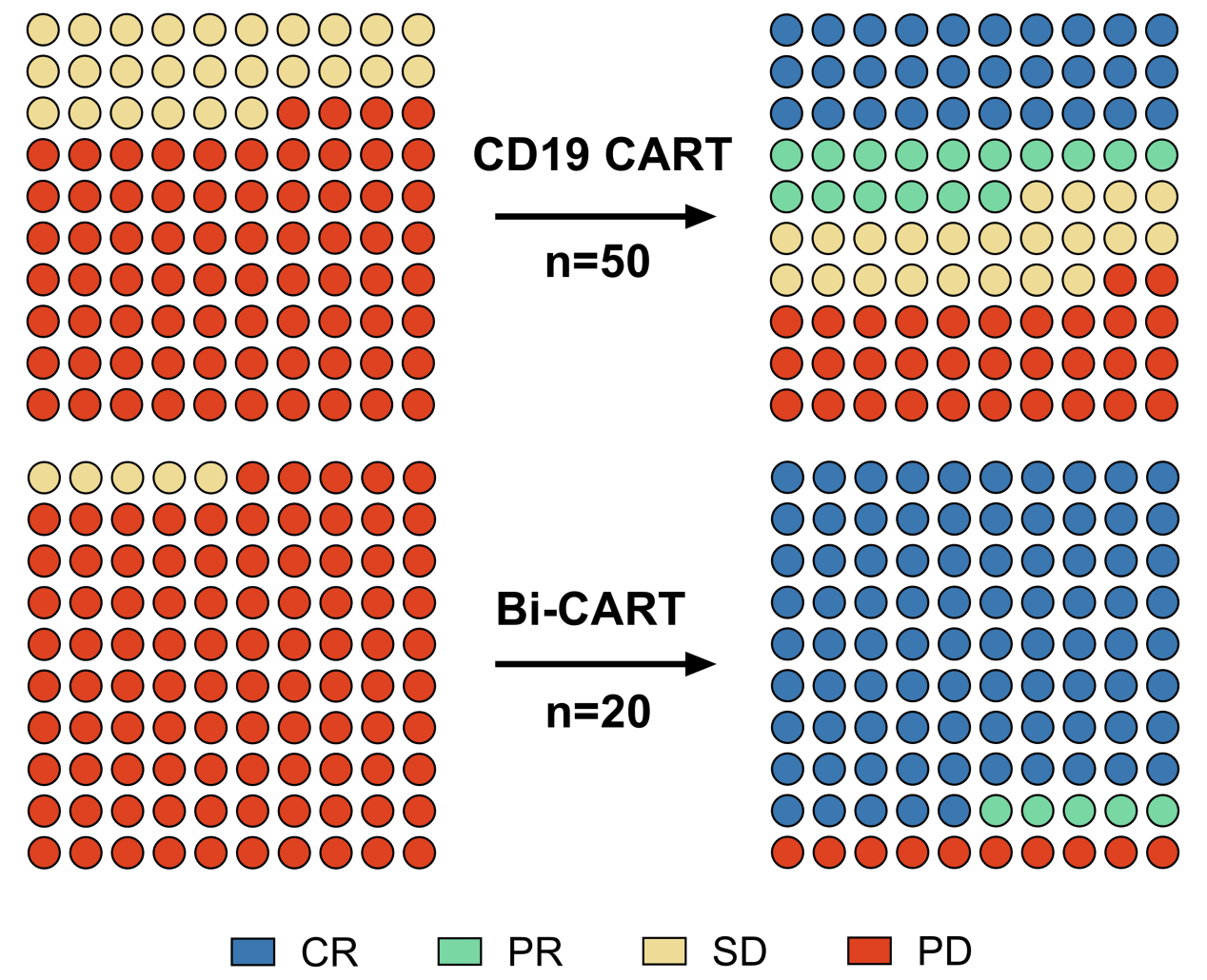

Supplement: Supplementary file 1 [file DataSheet1.docx]
