# Supplementary material for: Comparison of efficacy and adverse effects of CD19/20 CART versus CD19 single-target CART in R/R DLBCL: a single-center retrospective study
Source: Front Immunol. 2025 May 6;16:1582944. doi: 10.3389/fimmu.2025.1582944 (PMC12089104; doi:10.3389/fimmu.2025.1582944)
Supplement: Supplementary file 2 [file Table1.docx]

Supplementary Tabel 1. Subgroup analyses by treatment type

|  | **OR** | | | | **CR** | | | |
| --- | --- | --- | --- | --- | --- | --- | --- | --- |
|  | OR | 95%CI | P-value | P for interaction | OR | 95%CI | P-value | P for interaction |
| **Overall** | 1.08 | (0.33, 3.52) | 0.903 |  | 0.81 | (0.25, 2.61) | 0.719 |  |
| **Group, n** |  |  |  | 0.993 |  |  |  | 0.993 |
| CD19 CART, 50 | 0.65 | (0.18, 2.32) | 0.511 | 0.511 | 0.38 | (0.10, 1.41) | 0.147 | 0.147 |
| CD19/20 CART, 20 | 0 | (0, inf) | 0.997 | 0.997 | 0 | (0.25, 2.61) | 0.997 | 0.997 |

Supplementary Tabel 2. Baseline related to disease recurrence

| **Characteristics** | **Overall, N=70** | **No relapse, N=26** | **Relapse, N=44** | **P-value** |
| --- | --- | --- | --- | --- |
| **CART, n (%)** |  |  |  | 0.257 |
| CD19 | 50 (71) | 16 (62) | 34 (77) |  |
| CD19/20 | 20 (29) | 10 (38) | 10 (23) |  |
| **Gender, n (%)** |  |  |  | 0.666 |
| Male | 36 (51) | 12 (46) | 24 (55) |  |
| Female | 34 (49) | 14 (54) | 20 (45) |  |
| **Age at enrollment, n (%)** |  |  |  | 0.216 |
| <60y | 35 (50) | 16 (62) | 19 (43) |  |
| ≥60y | 35 (50) | 10 (38) | 25 (57) |  |
| **Hans classification, n (%)** |  |  |  | 0.969 |
| GCB | 20 (29) | 8 (31) | 12 (27) |  |
| N-GCB | 50 (71) | 18 (69) | 32 (73) |  |
| **Double expression, n (%)** |  |  |  | 0.650 |
| Yes | 28 (40) | 9 (35) | 19 (43) |  |
| No | 42 (60) | 17 (65) | 25 (57) |  |
| **Double/triple-hit, n (%)** |  |  |  | 0.289 |
| Yes | 4 (6) | 0 (0) | 4 (9) |  |
| No | 66 (94) | 26 (100) | 40 (91) |  |
| **Ann Arbor stage, n (%)** |  |  |  | 0.277 |
| Ⅰ-Ⅱ | 9 (13) | 5 (19) | 4 (9) |  |
| Ⅲ-Ⅳ | 61 (87) | 21 (81) | 40 (91) |  |
| **ECOG before infusion, n (%)** |  |  |  | 0.805 |
| 0 | 35 (50) | 14 (54) | 21 (48) |  |
| 1 | 35 (50) | 12 (46) | 23 (52) |  |
| **IPI score at enrollment, n (%)** |  |  |  | <0.001 |
| 0-2 | 35 (50) | 21 (81) | 14 (32) |  |
| 3-4 | 35 (50) | 5 (19) | 30 (68) |  |
| **Bulky disease, n (%)** |  |  |  | 0.243 |
| <7.5 cm | 62 (89) | 25 (97) | 37 (84) |  |
| ≥7.5 cm | 8 (11) | 1 (4) | 7 (16) |  |
| **Extra-nodual disease, n (%)** |  |  |  | >0.999 |
| 0-1 | 19 (27) | 7 (27) | 12 (27) |  |
| ≥2 organs | 51 (73) | 19 (73) | 32 (73) |  |
| **TP53, n (%)** |  |  |  | 0.007 |
| *(Missing)* | 5 (7) | 5 (19) | 0 (0) |  |
| WT | 41 (59) | 15 (58) | 26 (59) |  |
| Altered | 24 (34) | 6 (23) | 18 (41) |  |
| **Prior ASCT, n (%)** |  |  |  | >0.999 |
| Yes | 9 (13) | 3 (12) | 6 (14) |  |
| No | 61 (87) | 23 (88) | 38 (86) |  |
| **Prior radiotherapy, n (%)** |  |  |  | 0.502 |
| Yes | 18 (26) | 5 (19) | 13 (30) |  |
| No | 52 (74) | 21 (81) | 31 (70) |  |
| **Prior lines of therapy, n (%)** |  |  |  | 0.239 |
| 1-3 | 30 (43) | 14 (54) | 16 (36) |  |
| ≥4 lines | 40 (57) | 12 (46) | 28 (64) |  |
| **LDH level before infusion, n (%)** |  |  |  | 0.805 |
| ≤ULN (%) | 35 (50) | 14 (54) | 21 (48) |  |
| >ULN (%) | 35 (50) | 12 (46) | 23 (52) |  |

Supplementary Tabel 3. Subgroup analyses by IPI score and TP53 status

|  | **OR** | **95%CI** | **P-value** | **P for interaction** |
| --- | --- | --- | --- | --- |
| **Overall** | 0.47 | (0.16, 1.36) | 0.163 |  |
| **IPI score, n** |  |  |  | 0.205 |
| 0-2, 35 | 0.22 | (0.04, 1.25) | 0.088 |  |
| 3-4, 35 | 1.45 | (0.14, 15.04) | 0.753 |  |
| **TP53 status, n** |  |  |  | 0.870 |
| WT, 41 | 0.95 | (0.19, 4.71) | 0.952 |  |
| Altered, 24 | 0.77 | (0.11, 5.61) | 0.796 |  |

Supplementary Tabel 4. Univariate regression analysis of CRS

|  | **OR** | | | **CR** | | | **PFS** | | | **OS** | | |
| --- | --- | --- | --- | --- | --- | --- | --- | --- | --- | --- | --- | --- |
|  | OR | 95%CI | P-value | OR | 95%CI | P-value | HR | 95%CI | P-value | HR | 95%CI | P-value |
| **CRS, n** |  |  |  |  |  |  |  |  |  |  |  |  |
| No, 20 |  |  |  |  |  |  |  |  |  |  |  |  |
| Yes, 50 | 1.23 | (0.43, 3.50) | 0.701 | 0.79 | (0.28, 2.22) | 0.650 | 1.28 | (0.65, 2.54) | 0.477 | 1.32 | (0.62, 2.80) | 0.477 |
| **sCRS, n** |  |  |  |  |  |  |  |  |  |  |  |  |
| No, 48 |  |  |  |  |  |  |  |  |  |  |  |  |
| Yes, 2 | 0.66 | (0.04, 11.12) | 0.770 | 0 | (0, inf) | 0.999 | 0.89 | (0.12, 6.57) | 0.912 | 0.96 | (0.13, 7.14) | 0.969 |
| **CRS management, n** |  |  |  |  |  |  |  |  |  |  |  |  |
| Monitoring-only, 10 |  |  |  |  |  |  |  |  |  |  |  |  |
| NSAIDs-only, 33 | 1.17 | (0.27, 4.98) | 0.835 | 1.412 | (0.34, 5.94) | 0.638 | 0.65 | (0.29, 1.49) | 0.309 | 0.69 | (0.29, 1.69) | 0.420 |
| *Escalated therapy, 7 | 0.50 | (0.07, 3.55) | 0.488 | 0.60 | (0.08, 4.76) | 0.63 | 0.74 | (0.22, 2.46) | 0.618 | 1.10 | (0.32, 3.76) | 0.884 |

*Escalated therapy: NSAIDs with corticosteroids (n=5), NSAIDs with tocilizumab (n=1), NSAIDs with corticosteroids and tocilizumab (n=1).
